# Stabilizing the electrode–electrolyte interface for high-voltage Li‖LiCoO_2_ cells using dual electrolyte additives†

**DOI:** 10.1039/d5sc03120f

**Published:** 2025-06-19

**Authors:** Jiwei Ding, Chao Yang, Wenxi Hu, Xiaowei Liu, Anran Zhang, Deda Peng, Jin Han, Ya You

**Affiliations:** a State Key Laboratory of Advanced Technology for Materials Synthesis and Processing, Wuhan University of Technology Wuhan 430070 P. R. China youya@whut.edu.cn; b International School of Materials Science and Engineering, School of Materials Science and Microelectronics, Wuhan University of Technology Wuhan 430070 P. R. China jinhan@whut.edu.cn; c Hubei Longzhong Laboratory, Wuhan University of Technology Xiangyang Demonstration Zone Xiangyang 441000 China

## Abstract

Rechargeable Li‖LiCoO_2_ batteries are attractive due to their high energy density. However, the growth of lithium dendrites and the degradation of cathode at high potential hinder their practical application. Herein, we propose an advanced fluorinated carbonate-based electrolyte consisting of *trans*-4,5-difluoro-1,3-dioxolan-2-one (DFEC) and tri-(trimethylsilyl) phosphite (TMSPi) as dual additives to construct a stable interface on both the anode and cathode. The results show that DFEC can promote the formation of a stable solid-electrolyte-interface (SEI) layer on the lithium anode to inhibit the growth of dendrites. Additionally, TMSPi is conducive to the production of an inorganic-rich cathode–electrolyte interface (CEI) layer on the LiCoO_2_ cathode to inhibit the dissolution of cobalt. Finally, the Li‖LiCoO_2_ cells with this electrolyte could obtain an initial capacity of 211.6 mAh g^−1^ (846.4 Wh kg^−1^, active substances based on cathodes) at 1C (1C = 274 mA g^−1^) with a high-capacity retention of 81.6% after 200 cycles at a high upper cut-off voltage of 4.6 V. This work provides valuable insights into the development of electrolytes for high-voltage Li‖LiCoO_2_ cells.

## Abstract 2

This work reports dual additives for high-voltage Li/LiCoO_2_ cells, which could simultaneously stabilize the electrode–electrolyte interfaces on both cathode and anodes.

## Graphical abstract

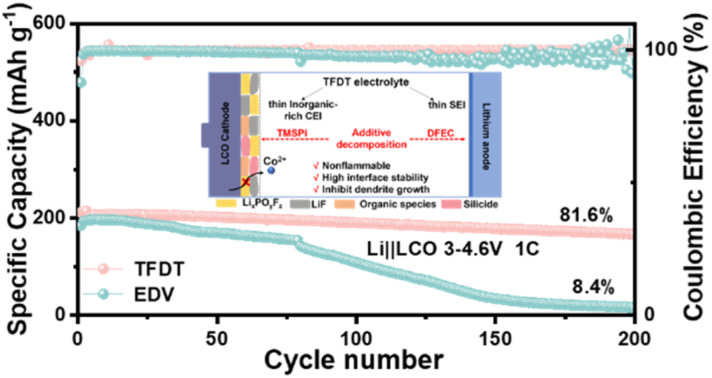

## Introduction

Rechargeable Li‖LiCoO_2_ cells have broad application prospects because of their high energy density. Unfortunately, LiCoO_2_ cathodes can only deliver a relatively low reversible capacity (∼140 mAh g^−1^) at the upper cut-off voltage of 4.20 V, despite their high theoretical capacity (274 mAh g^−1^).^1–3^ Raising the upper cut-off voltage of LiCoO_2_ has been proven to be an effective way to increase the practical capacity. However, once the upper cut-off voltage approaches 4.55 V, the electrolyte can suffer from severe decomposition in the absence of a stable interface on the cathode, leading to continuous capacity degradation of LiCoO_2_.^4–6^ Additionally, lithium metal anodes are attractive due to their outstanding theoretical capacity (3860 mAh g^−1^) and low redox potential (−3.04 V *vs.* SHE).^7–11^ However, lithium-dendrite growth and the accumulation of dead lithium lead to a short cycle life and low Coulomb efficiency, hindering the practical application of lithium metal anodes. To address the above-mentioned issues with both the cathode and anode of Li‖LiCoO_2_ cells, electrode/electrolyte interface engineering seems to be an effective strategy.^12–17^

Electrode/electrolyte interface engineering can be mainly classified into artificial interface and electrolyte engineering.^18–21^ However, artificial interfaces often require a relatively complex preparation process, which is not conducive to large-scale production. In contrast, electrolyte engineering through the use of additives is a simple and feasible approach. For instance, 2,3-dimethylmaleic anhydride was used as a film-forming additive for constructing a dense and uniform CEI film on an LiCoO_2_ cathode, thereby enhancing the interface stability of the LiCoO_2_ cathode.^22^ 2-Fluoro-5-iodopyridine has been proposed as a film-forming additive for lithium metal anodes, and addressed the issue of dendrite growth on lithium metal anodes.^23^ However, these electrolyte formulations can only solve the problem with the LiCoO_2_ cathode or the lithium metal anode individually.^24–26^ Therefore, there is an urgent need to explore novel electrolyte formulations capable of simultaneously stabilizing both the electrodes.

In this work, we adopted a dual-additive strategy and selected DFEC with a low lowest unoccupied molecular orbital (LUMO) energy level and TMSPi with a high highest occupied molecular orbital (HOMO) energy level as the film-forming additives for the lithium metal anode and the LiCoO_2_ cathode, respectively. The results show that DFEC can contribute to the generation of a stable SEI layer on the lithium metal anode to inhibit the lithium-dendrite growth. TMSPi can induce the formation of an inorganic-rich CEI layer on the LiCoO_2_ cathode to inhibit the dissolution of cobalt and enhance the cycling stability. Finally, the proposed electrolyte enabled Li‖LiCoO_2_ cells with an initial capacity of 211.6 mAh g^−1^ at 1C and a high-capacity retention of 81.6% after 200 cycles at a high upper cut-off voltage of 4.6 V.

## Results and discussion

### Characterization of the electrolyte

**Fig. 1:**
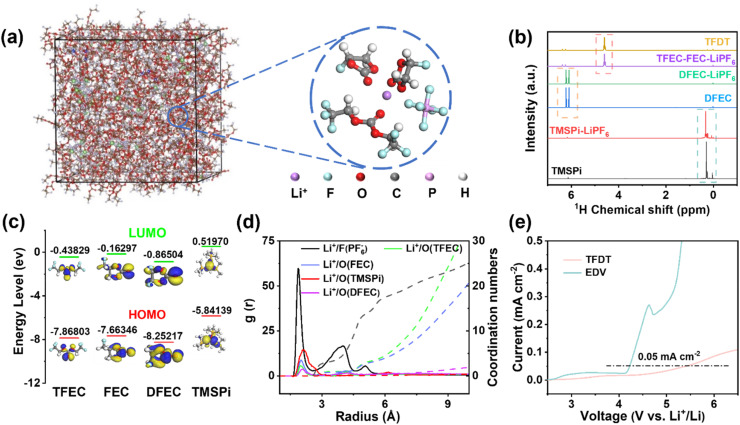
(a) Snapshot and locally enlarged solvation structure acquired from MD simulations of the electrolyte TFDT. (b) ^1^H NMR of various solvents and electrolytes. (c) LUMO/HOMO energy level diagram for the molecules TFEC, FEC, DFEC, and TMSPi. (d) RDF results for Li^+^ acquired from MD simulations for TFDT. (e) Electrochemical stability window of the electrolytes EDV and TFDT on the anodic side determined by linear sweep voltammetry (LSV) in a coin cell comprising stainless steel as the working electrode and Li metal as the counter electrode at a scanning rate of 0.1 mV s^−1^.

The solvation structure of the electrolyte TFDT (1 M LiPF_6_ dissolved in a blend of bis(2,2,2-trifluoroethyl) carbonate (TFEC), fluoroethylene carbonate (FEC), DFEC and TMSPi (4 : 1 : 0.05 : 0.05, vol%)) was revealed using molecular dynamics (MD) simulation. The selected snapshots and locally enlarged structures (Fig. 1a) show that the Li^+^ cations are closely coordinated with TFEC, FEC, and PF_6_^−^ anions. Additionally, the snapshot results show that the additives DFEC and TMSPi are not involved in the solvation structure. This conclusion was further supported by the subsequent nuclear magnetic resonance (NMR) characterization. In the ^1^H NMR spectra (Fig. 1b), no peak shift was observed between the spectra of TFDT with and without the additives DFEC and TMSPi, indicating that these two additives are not involved in the solvation structure. The local coordination environment around the Li^+^ cations was obtained by analyzing the radial distribution function (RDF) (Fig. 1d). The average coordination number of the Li^+^ cations with PF_6_^−^, TFEC and FEC was 3.3, 0.97, and 0.95, respectively. However, the average coordination number with the additives DFEC and TMSPi is negligible, indicating that they are not involved in the solvation structure.^27–29^

To investigate the redox reactivity of the included solvents and additives in the electrolytes, the corresponding HOMO and LUMO were calculated and analyzed using frontier molecular orbital theory (Fig. 1c). Due to the introduction of fluorine, the fluorinated organic solvents TFEC and FEC show higher LUMO levels and lower HOMO levels than the commercial carbonate solvent, indicating their superior oxidation stability. Notably, the additive DFEC possesses a lower LUMO level than the main solvents TFEC and FEC, indicating that DFEC can be preferentially reduced on the lithium metal anode before TFEC. Additionally, the HOMO level of TMSPi (−5.84139 eV) is higher than that of TFEC and FEC (−7.66346 eV), indicating that TMSPi is preferentially oxidized before TFEC and FEC on the LiCoO_2_ cathode. Thus, the additives DFEC and TMSPi could potentially be decomposed to produce an interface on the lithium metal anode and LiCoO_2_ cathode simultaneously. To evaluate the electrochemical stability of the electrolytes, LSV was conducted at a scanning rate of 0.1 mV s^−1^ on the anodic side in a coin cell containing stainless steel as the working electrode and Li metal as the counter and reference electrode. 1 M LiPF_6_ dissolved in a blend of ethylene carbonate (EC), diethyl carbonate (DEC) and vinylene carbonate (VC) (3 : 7 : 0.1, vol%)—denoted as EDV—was chosen as the reference electrolyte. The results in Fig. 1e show that the current rises sharply in EDV when the potential is higher than 3 V (*vs.* Li/Li^+^). In comparison, for TFDT, the current remains relatively stable until the potential is higher than 5.0 V, demonstrating its prominent anodic electrochemical stability.^30,31^

### Electrochemical performance and interface characterization of the LiCoO_2_ cathode

**Fig. 2:**
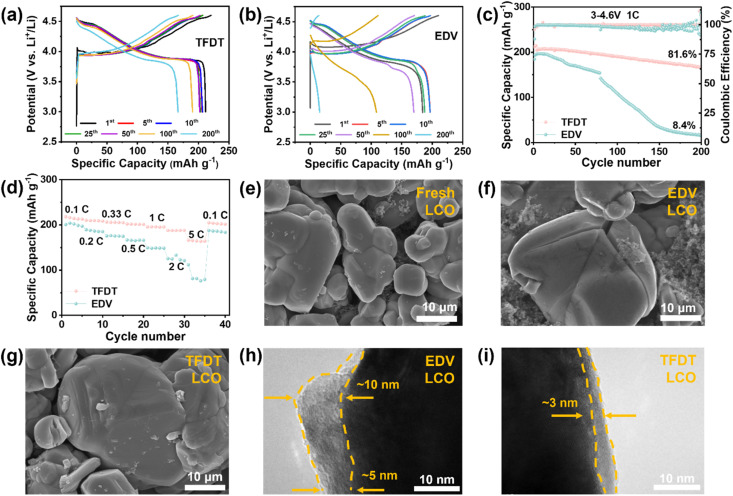
Galvanostatic charge and discharge curves of Li‖LiCoO_2_ cells with the electrolytes TFDT (a) and EDV (b) at different cycles. (c) Cycle performance of Li‖LiCoO_2_ cells with both electrolytes at 1C rate. (d) Rate performance of Li‖LiCoO_2_ cells with EDV and TFDT. SEM images of fresh LiCoO_2_ (e) and the LiCoO_2_ cathodes cycled in EDV (f) and TFDT (g) for 50 cycles at 1C with an upper charge cut-off voltage of 4.6 V. TEM images of the LiCoO_2_ cathodes cycled in EDV (h) and TFDT (i) under the same measurement conditions.

To further evaluate the effect of the electrolytes on the electrochemical performance, the cycle performance of Li‖LiCoO_2_ cells with the two electrolytes was measured with an upper cut-off voltage 4.6 V at 1C after it had been activated at 0.1C for the first three cycles (Fig. 2a and b). An initial discharge capacity of 195.9 mAh g^−1^ was delivered by Li‖LiCoO_2_ cells with the electrolyte EDV, while the current rapidly decreased to 108.57 mAh g^−1^ after the 100^th^ cycle. In comparison, Li‖LiCoO_2_ cells with TFDT exhibited an initial capacity of 211.6 mAh g^−1^ and maintained a specific capacity of 190.37 mAh g^−1^ after the 100th cycle. The corresponding coulombic efficiency for the Li‖LiCoO_2_ cells with the electrolytes TFDT and EDV is 95.94% and 87.8%, respectively. Notably, TFDT endowed the cell with a high-capacity retention of 81.6% after 200 cycles, which was much higher than that achieved using EDV (7%) (Fig. 2c). Throughout the entire 200 cycles, the coulombic efficiency of the cell with the TFDT was consistently higher than that of the EDV cell. The reason behind the quite different cycling performances of the electrolytes EDV and TFDT was elucidated using XRD (Fig. S1†). Although the LiCoO_2_ electrode cycled in the EDV has similar characteristic peaks to the pristine LiCoO_2_ electrode, after cycling in TFDT, the peak intensities of the 003 and 012 crystal planes of the LiCoO_2_ were reduced to a much lesser extent compared to those cycled in EDV (Table S1†), indicating that the CEI layer generated in TFDT maintains the structural stability of LiCoO_2_. In addition, the first cycle of the cell with TFDT shows obvious fluctuations in the curve when the voltage is around 4.0 V, while these disappear in the second cycle. This phenomenon can likely be attributed to the decomposition of the electrolyte additive in the first cycle (Fig. S2†) and is not present in EDV (Fig. S3†). However, the charging and discharging curves suffer dramatic variations. A high average discharge potential of 4.0 V can be delivered by Li‖LiCoO_2_ cells with TFDT throughout 200 cycles, while the value drops from 4.0 V to 3.5 V with EDV (Fig. S4†).

The rate performance of Li‖LiCoO_2_ cells using EDV and TFDT was evaluated under cycling from 0.1C to 5C in the range of 3.0–4.6 V (Fig. 2d). The Li‖LiCoO_2_ cell with TFDT can attain capacities of 214.2, 209.4, 206.1, 201.9, 195.7, and 187.3 mAh g^−1^ at 0.1, 0.2, 0.33, 0.5, 1, 2, and 5C, respectively. When the current density is returned to 0.1C, the cell can recover to 202.9 mAh g^−1^ (94.7% retention rate) and maintain stable cycling performance in subsequent cycles. In comparison, the capacity of the Li‖LiCoO_2_ cell with EDV was much lower than that of the TFDT at the corresponding current density, indicating the enhanced lithium-ion transport dynamics endowed by TFDT. Furthermore, a constant current intermittent titration test (GITT) was conducted to measure the Li^+^ diffusion coefficient when using the electrolytes TFDT and EDV (Fig. S5†). The results indicate that the Li^+^ diffusion coefficient in the Li‖LiCoO_2_ cell with TFDT is higher than that in the Li‖LiCoO_2_ cell with EDV.

The surface morphology of the LiCoO_2_ cathode was probed using SEM after 50 cycles in the different electrolytes at 3–4.6 V, as shown in Fig. 2e–g. The results show that large cracks appear on the surface of the LiCoO_2_ particles after 50 cycles in EDV (Fig. 2f), which are not found for the fresh LiCoO_2_ electrode (Fig. 2e) or the LiCoO_2_ electrode cycled in TFDT (Fig. 2g). To further inspect the LiCoO_2_ electrode interface, transmission electron microscopy (TEM) was performed on the LiCoO_2_ cathodes cycled in EDV and TFDT. It was found that an inhomogeneous CEI layer with a varying thickness (5–10 nm) was formed on the LiCoO_2_ cathode in EDV (Fig. 2h), whereas a dense and thin (3–4 nm) CEI layer was generated on the LiCoO_2_ cathode in TFDT (Fig. 2i). The above results could indicate that the severe degradation of LiCoO_2_ particles in EDV is related to the absence of a stable CEI layer, while the structural stability of LiCoO_2_ particles in TFDT benefits from the protection of a robust CEI layer.

#### Characterization of the Li metal anode

**Fig. 3:**
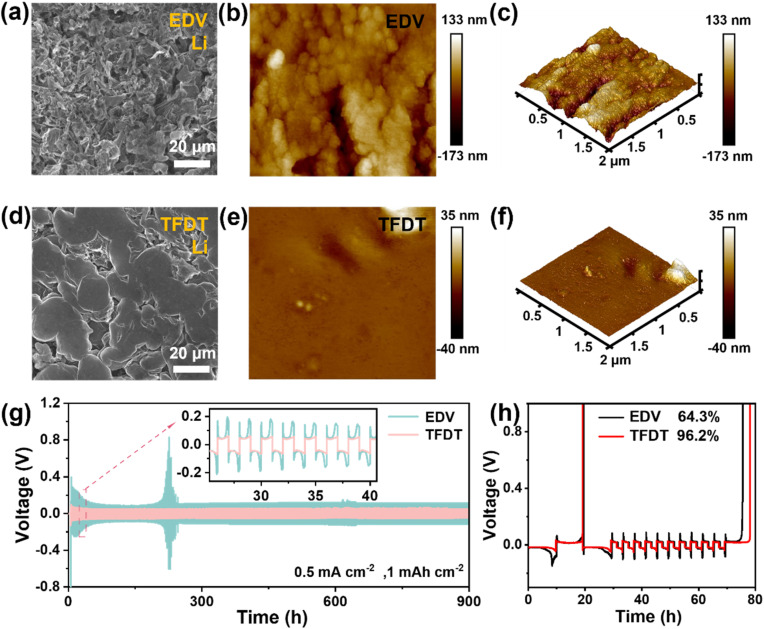
SEM images of lithium metal anodes cycled in the electrolytes EDV (a) and TFDT (d) after 50 cycles at 1C with an upper charge cut-off voltage of 4.6 V in Li‖LiCoO_2_ coin cells. AFM images of lithium metal anodes cycled in EDV (b and c) and TFDT (e and f) under the same measurement conditions. (g) Plating/stripping voltage profiles of the Li‖Li symmetrical cells in EDV and TFDT. (h) Voltage curves of plating/stripping for Li‖Cu cells with EDV and TFDT.

To inspect the lithium-dendrite growth on lithium anodes in Li‖LiCoO_2_ coin cells, the morphology of Li metal anodes cycled in different electrolytes was examined using scanning electron microscopy (SEM) after 50 cycles at 3–4.6 V. Irregular multi-dendrite porous structures were clearly observed on the Li anode with the baseline electrolyte EDV (Fig. 3a). This porous structure implies the production of dead lithium and lithium dendrites in the electrolyte. In contrast, the morphology of the plated Li is relatively flat without obvious dendrites in TFDT (Fig. 3d), indicating that the uniform lithium deposition induced by the SEI layer was derived from the TFDT. The morphology of the lithium metal anode surface after cycling in the different electrolytes was further evaluated using atomic force microscopy (AFM) characterization. As shown in Fig. 3b and c, the surface of lithium metal cycled in EDV is relatively rough. The longitudinal profile of the lithium metal surface is located between −173 nm and 133 nm, and the root mean square roughness (*R*_q_) and arithmetic average roughness (*R*_a_) are 38.7 nm and 28.7 nm (Table S2†), respectively. In contrast, the surface of the lithium metal cycled in TFDT is relatively smooth, as shown in Fig. 3e and f. The corresponding longitudinal profile of the surface is located between −40 nm and 35 nm, and the roughness results of *R*_q_ and *R*_a_ are 3.95 nm and 2.01 nm, respectively. In short, TFDT effectively suppressed the dendrite growth and guided a flat lithium deposition by generating a robust SEI.

Li/Li and Li/Cu cells were assembled and measured to evaluate the effect of the electrolyte TFDT on lithium plating and stripping. In the symmetric Li/Li cells setup at a current density of 1 mAh cm^−2^, TFDT endowed the cell with a relatively low polarization voltage (∼51 mV) and a long cycle life of 900 h (Fig. 3g), while the commercial electrolyte EDV resulted in a high polarization voltage (∼118 mV) with an unstable phenomenon. The Auerbach method was used to detect the stability of electrolytes, and the average coulomb efficiency (CE) during plating/stripping was recorded for Li/Cu asymmetric cells, as shown in Fig. 3h.^32^ TFDT endowed the Li/Cu cells with a higher average CE (96.2%) than EDV (64.3%), indicating that TFDT is more compatible with lithium metal. The exchange current density (*J*_0_) of Li‖LiCoO_2_ cells with the electrolytes TFDT and EDV was assessed using Tafel curves during the plating/stripping process at 20 °C. The results show that TFDT generates a higher *J*_0_ than EDV (0.116 *vs.* 0.007 mA cm^−2^) (Fig. S6†), indicating faster kinetics and better reversibility for lithium plating/stripping in TFDT.^33^

#### Interfacial chemistry

**Fig. 4:**
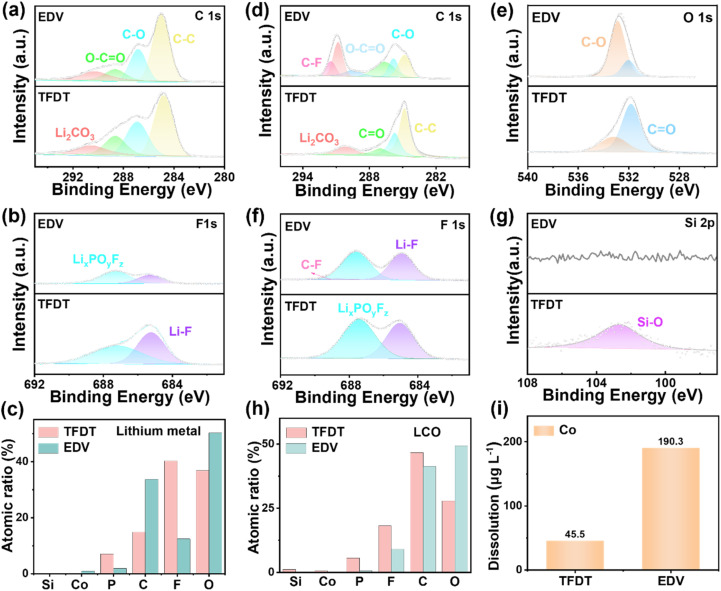
High-resolution C 1s (a) and F 1s (b) XPS spectra with deconvolution for lithium metal anodes after 50 cycles in the electrolytes EDV and TFDT. (c) Relative contents of the elements in the SEI layer formed on the surfaces of the lithium metal anodes in the different electrolytes. High-resolution C 1s (d), O 1s (e), F 1s (f), and Si 2p (g) XPS spectra with deconvolution for LiCoO_2_ cathodes after 50 cycles in the electrolytes EDV and TFDT. (h) Relative contents of the elements in the CEI layer on the surface of the LiCoO_2_ cathode. (i) Amount of Co that escaped from the LiCoO_2_ cathodes after 50 cycles in the different electrolytes.

To further reveal the influence of the TFDT electrolyte on the interface, X-ray photoelectron spectroscopy (XPS) analysis was first performed on the lithium metal anode (Fig. 4a and b). The C 1s spectra in Fig. 4a reveal a C–C peak at 284.8 eV, which was assigned to the conductive carbon. The C–O peak at 285.6 eV and O–C=O peak at 289.3 eV originate from Li_2_CO_3_ as a result of the decomposition of the organic carbonate solvents. In the F 1s spectra (Fig. 4b), the lithium metal anode in TFDT reveals a stronger signal of inorganic LiF (284.8 eV) and Li_*x*_PO_*y*_F_*z*_ (284.8 eV) than that of EDV. The intensity of all elements was normalized to evaluate their relative contents in the SEI layer (Fig. 4c). The relative proportions of F and P in the SEI formed in TFDT are significantly higher than those in the SEI formed in EDV; in turn, the proportions of C and O in the SEI formed in TFDT were lower. This indicates that the SEI layer formed on the lithium metal anode in TFDT contains inorganic-rich substances (LiF and Li_*x*_PO_*y*_F_*z*_), thereby enhancing the interface stability of the lithium metal anode.

To probe the interface chemistry on the LiCoO_2_ cathodes cycled in the electrolytes TFDT and EDV, the XPS spectra for the LiCoO_2_ cathode in a charged state (4.6 V) are presented in Fig. 4d–g. In the C 1s spectra (Fig. 4d), the spectra peak at 285.6 eV, 287.1 eV, and 289.3 eV are ascribed to C–O, C=O, and O–C=O functional groups, respectively. The peaks at 290.1 eV and 291.4 eV were potentially due to Li_2_CO_3_ and polyvinylidene fluoride (PVDF).^4^ It is speculated that the CEI on the LiCoO_2_ surface in EDV is much richer in organic species than the TFDT one. Regarding the O 1s spectra in Fig. 4e, the peaks at 531.8 eV and 533.0 eV are assigned to C=O and C–O functional groups, respectively.^34^ When comparing the inorganic fluorine-containing species in the CEIs (Fig. 4f), the CEI formed in TFDT shows a higher signal for Li_*x*_PO_*y*_F_*z*_, which is mainly due to the decomposition of the additive TMSPi during the cycling process. Furthermore, the Si–O peak at 103 eV for the CEI on the LiCoO_2_ cycled in TFDT is ascribed to the decomposition of the additive TMSPi (Fig. 4g). The Si–O bond has a relatively high bond energy, which can form a stable chemical adsorption layer on the electrode surface and enhance the mechanical strength of the interface, maintaining the stability of the interface.^35^ The XPS spectra were normalized to calculate the relative proportions of different substances in the CEI (Fig. 4h). The relative proportions of C, F, P and Si in the CEI on the LiCoO_2_ cathode in TFDT were significantly higher than those on LiCoO_2_ cathode with EDV, while the proportion of O was lower for the LiCoO_2_ with TFDT. These collective findings indicate that the CEI formed in TFDT mainly contains inorganic substances, which is beneficial for stabilizing the material structure of the LiCoO_2_ cathode.^36^ In addition, the effective influence of the CEI interface on the LiCoO_2_ was investigated using inductively coupled plasma optical emission spectroscopy (ICP), as shown in Fig. 4i. It was found that the amount of cobalt leached in TFDT was 45.5 μg L^−1^, which was just 23.9% the amount leached in EDV (190.3 μg L^−1^), indicating the enhanced structural stability of the LiCoO_2_ cathode in TFDF.

#### Thermal safety test for EDV and TFDT electrolytes

**Fig. 5:**
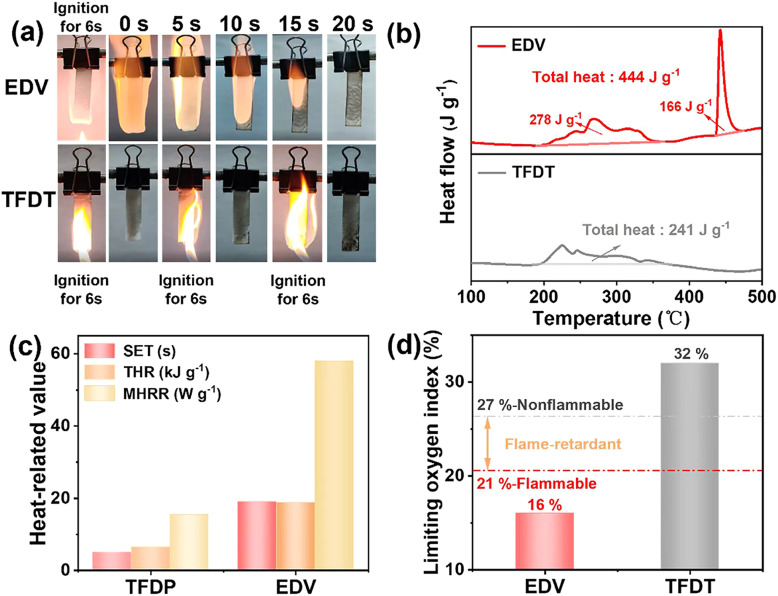
(a) Ignition tests for EDV and TFDT electrolytes. (b) DSC profiles of charged-state LiCoO_2_ (4.6 V) mixed with EDV or TFDT in a weight ratio of 6.5 : 3.5 at a scan rate of 5 °C min^−1^ between 100 and 500 °C. (c) Self-extinguishing time (SET), time to ignite (TTI), and total heat release (THR) of EDV and TFDT as determined using a cone calorimeter. All samples were exposed to heat radiation until they had thoroughly ignited. (d) Limiting oxygen indices of EDV and TFDT.

Organic electrolytes under deep delithiation will undergo intense exothermic reactions; therefore, thermal safety is crucial for lithium-ion cells.^37,38^ The flammability of the electrolytes was assessed using a conventional ignition test, as shown in Fig. 5a. The TFDT electrolyte could not be ignited after being exposed to the flame of a methane lighter for 6 s, nor was it ignited in two subsequent attempts, while the EDV electrolyte caught fire rapidly and burned violently until the electrolyte had burned off. Differential scanning calorimetry (DSC) was used to measure the thermal abuse tolerance of the electrolytes when paired with a charged-state LiCoO_2_ electrode at 4.6 V (Fig. 5b). It can be seen that the LiCoO_2_/EDV mixture showed an exothermic reaction with a total exothermic heat of 444 J g^−1^. However, the total heat release of the LiCoO_2_/TFDT mixture was 241 J g^−1^, which was 54.3% that of the LiCoO_2_/EDV mixture. These results indicate that TFDT can reduce the heat production of exothermic reaction for the electrode and electrolyte mixture.

To further investigate the heat-related behavior associated with these electrolytes, a cone calorimeter was used to collect some parameters of the combustion process, such as the self-extinction time (SET), total heat release (THR), and maximum heat release rate (MHRR). All samples were exposed to a constant thermal radiation value of 25 kW m^−2^ until fully ignited. The TFDT electrolyte has a lower MHRR (15.4 W g^−1^*vs.* 58.1 W g^−1^), lower SET (5 s *vs.* 19 s) and lower THR (6.54 kJ g^−1^*vs.* 18.78 kJ g^−1^) than EDV electrolyte, as shown in Fig. 5c. In addition, the gas produced during the combustion of TFDT has a higher CO concentration and lower CO_2_ content than that of EDV, which indicates the incomplete combustion and notable flame retardance of TFDT (Table S3†). To quantify the flammability of the electrolytes, the limiting oxygen index (LOI) test was used to further measure their flammability. As shown in Fig. 5d, TFDT has a much higher LOI (32%) than TFDT (16%). Based on the classification zones in Fig. 5d, TFDT has flame- retardance characteristics, while EDV is flammable. In summary, TFDT has better flame retardance and self-extinguishing characteristics compared with EDV. Additionally, the lower heat release during combustion may be related to the higher concentration of fluorine-containing solvents in TFDT.

## Conclusions

In summary, a novel electrolyte containing dual additives was designed, and its corresponding solvation structure was studied. The electrolyte has properties that optimize the formation of electrolyte interfaces. The TMSPi additive enhances the interface stability of the LiCoO_2_ cathode by forming stable CEI layers, thereby further preventing the structural collapse of LiCoO_2_ and the leaching of cobalt under high operation potential. Additionally, the additive DFEC generates an inorganic-rich SEI layer on the surface of the lithium metal anode and inhibits the growth of lithium dendrites. Therefore, the tailored electrolyte endowed Li/LiCoO_2_ cells with an initial capacity of 211.6 mAh^−1^ (846.4 W h kg^−1^) and a high-capacity retention of 81.6% after 200 cycles at a high cut-off voltage of 4.6 V. This work broadens the perspective on electrolyte development for high-voltage Li/LiCoO_2_ cells through the use of electrolyte additives.

## Author contributions

Y. Y. and J. H. conceived and supervised the project; J. D. designed the experiments; J. D., W. H., and D. P. conducted the experiments; X. L. and A. Z. conducted the DFT calculations and MD simulations. J. D. and C. Y. conducted the characterizations and analysis. J. D. drafted the manuscript with the help of Y. Y. and J. H.; all authors agreed on the manuscript and approved submission.

## Conflicts of interest

There are no conflicts to declare.

## Supplementary Material

SC-OLF-D5SC03120F-s001

## Data Availability

The data supporting this article have been included as part of the ESI.†
