# Supplementary material for: Stabilizing the electrode–electrolyte interface for high-voltage Li‖LiCoO2 cells using dual electrolyte additives
Source: Chem Sci. 2025 Jun 19;16(30):13723–30. doi: 10.1039/d5sc03120f (PMC12216582; doi:10.1039/d5sc03120f)
Supplement: SC-016-D5SC03120F-s001 [file SC-016-D5SC03120F-s001.pdf]

Supplementary Information (SI) for Chemical Science.
This journal is © The Royal Society of Chemistry 2025

Supplementary Information (SI) for Chemical Science.
This journal is © The Royal Society of Chemistry 2025

Supporting Information

Stabilizing electrode-electrolyte interface for high-voltage Li||$LiCoO_2$ cells by dual electrolyte additives

Jiwei Ding [a], Chao Yang [a], Wenxi Hu [a], Wenxi Hu [a], Xiaowei Liu [a], Anran Zhang [a], Deda Peng [a], Jin Han [b,*], Ya You [a, b, c, *]

a. State Key Laboratory of Advanced Technology for Materials Synthesis and processing, Wuhan University of Technology, Wuhan 430070, P. R. China

b. International School of Materials Science and Engineering, School of Materials Science and Microelectronics, Wuhan University of Technology, Wuhan 430070, P. R. China

c. Hubei Longzhong Laboratory, Wuhan University of Technology, Xiangyang Demonstration Zone, Xiangyang 441000, China

Corresponding Author

Jin Han*, Ya You*

E-mail: jinhan@whut.edu.cn, youya@whut.edu.cn.

1.Experimental section

Materials

Lithium cobalt oxide (LCO) (98%) was purchased from Tianjin Guoan Mengguli New Materials Science&Technology Co.,Ltd, and used directly for all measurements without any processing. Trans-4,5-difluoro-1,3-dioxolan-2-one (DFEC) (99.9%), bis(2,2,2-trifluoroethyl) carbonate (TFEC) (99.9%), fluoroethylene carbonate (FEC) (99.9%) and lithium hexafluorophosphate ($LiPF_6$) (99.9%)were purchased from Duoduo-Chem. Tri-(trimethylsilyl) phosphite (TMSPi) (95%) was hased from Aladdin. All the chemicals and reagents were used without further purification.

Preparation of electrolyte and coin assembly: All operations are carried out under an Ar-filled glove box with an index of O_2/H_2O < 1 ppm. (1) EDV-base electrolyte: 1.0 M $LiPF_6$ salt was dissolved in blended ethylene carbonate (EC) and diethyl carbonate (DEC) solvents with a volume ratio of 3:7. Then, add 1 vol.% vinylene carbonates into above mixed solvent, and stir until transparent to obtain EDV electrolyte. (2) TFDT electrolyte (1 M): 1M $LiPF_6$ solution was prepared by bis(2,2,2-trifluoroethyl) carbonate (TFEC) as the main solvent and fluoroethylene carbonate (FEC) as the cosolvent (volume ratio: 4:1). Afterwards, equal volume of trans-4,5-Difluoro-1,3-dioxolan-2-one (DFEC) and tri-(trimethylsilyl) phosphite (TP) was added into above solution to form a uniform and stable electrolyte. The $LiCoO_2$ electrode was obtained by coating the slurry of $LiCoO_2$ powders, acetylene black and poly (vinylidene difluoride) with a weight ratio of 8: 1: 1 in N-methyl-2-pyrrolidone solvent onto an aluminum current collector. Dry the slurry for 6 h at 80 °C and punch into disks with diameter of 12 mm. CR2025 coin cells with different electrolytes were encapsulated according to the sequence of cathode, separator, and anode in a glove box for further testing. The mass loading is about 2.5~ 5 mg cm^{-2}.

Electrochemical characterization: The voltage range was set to 3.0 - 4.60 V for the Li||LCO cells. An electrochemical workstation (Corrtest, CS3104, Wuhan) was used to evaluate the AC impedance, electrochemical stability window of the electrolyte, and

the oxidation-reduction behavior of Li||LCO cell. Linear sweep voltammetry (LSV) was carried out on a coin cell with a scan rate of 1 mV s^{-1}. The charge and discharge tests were operated by using LAND system. The Li||LCO cells were charged and discharged at 1/3 C and 1 C (1 C = 274 mA h g^{-1}) between 3.0 - 4.6 V to assess electrochemical performance. The cell was cycled at continuous 8 cycles with various currents of 0.1 C, 0.2 C, 0.33 C, 0.5 C, 1 C, 2 C, 5 C, 0.1 C respectively for rate capability.

Combustion test of the electrolyte: It is mainly conducted through two types of combustion experiments. (1) **Ignition tests:** In a typical measurement process, electrolyte was soaked into a porous nonflammable matrix, constant area glass fiber (1.5 cm * 6 cm). After holding the ignition for 6 s, the time was recorded until completely extinguished after removal of fire source. Time to ignition (TTI) and self-extinguishing time (SET) are used to measure the flammability of electrolyte. They are generally normalized to the mass and represented by s g^{-1}. (2) **Cone calorimeter experiment:** The sample is placed on the sample support of the loading table, and the outer surface is exposed to the thermal radiation of the conical heater with rated heat flow intensity of 25 kW m^{-2}. The volatilized combustible gas is ignited by an igniter after heated, and various parameters are obtained through the detector.

2.Materials characterization

Interface testing： A field-emission scanning electron microscope (SEM, JEOL-7100F) was used to observe the surface morphology of cathode and anode at an acceleration voltage of 10 kV. The high-resolution transmission electron microscope (HR-TEM, JEOL JEM-2100F) was utilized to observe the CEI layer. The surface information of the produced samples was determined by X-ray photoelectron spectroscopy (XPS, ESCALAB 250Xi). The atomic force microscopy (AFM) is used to observe the appearance of the anode surface. The crystal structure of prepared samples was analyzed using the X-ray diffraction (XRD) technique, specifically employing a Bruker D8 Advance X-ray diffractometer with Cu Kα radiation (2θ from 10 to 80°).

DSC measurement: Differential scanning calorimetry (DSC) measurement were performed on a thermal analysis system (Simultaneous Thermal Analysis, STA449F3, NETZSCH). Delighted $LiCoO_2$ cathode with around 25 mg mass loading were retrieved from cells after charged to 4.6V vs. Li^+/Li, and then repeatedly rinsed with dimethyl carbonate (DMC) for three times to absolutely remove the residual electrolyte. After overnight drying, the electrode powder was mixed with selected electrolyte solutions and placed into a 100 μL high-pressure gold DSC capsule with a seal. The DSC profiles were recorded between 100 and 500 °C with a scan rate of 5 °C min^{-1}. An empty stainless-steel vessel was used as a reference in all tests. The calculated heat generation during the measurement is expressed in the manuscript with the unit J g^{-1}, and the latter part of which (g) refers to the mass of the active cathode materials.

Solvation structures characterization: Electrolyte solvation structures were characterized using nuclear magnetic resonance spectroscopy (NMR, Avance III HD 500 MHz). The ^1H NMR spectra were acquired in the case of deuterated acetone with TMS used as an internal reference.

Molecular dynamics (MD) simulations: The molecular dynamics (MD) simulations were conducted by using the Forcite package in Materials Studio. The COMPASS

forcefield was applied for the all simulations. The summation method of Ewald was set for describing the electrostatic and van der Waals interactions in the systems. The Nose themostat and Berendsen barostat were selected for controlling the temperature and pressure. All electrolyte supercells were firstly annealed with the temperature ranging from 300 to 500 K for 5 cycles. The obtained supercells were then pre-equilibrated by using the NPT ensemble with the time step of 0.5 fs and total time of 1000 ps. The supercells were further equilibrated by applying the NVT ensemble with the same time step and equilibrium time. The statistical analyses were performed at the second half of this equilibration process.

3. Supplementary figures

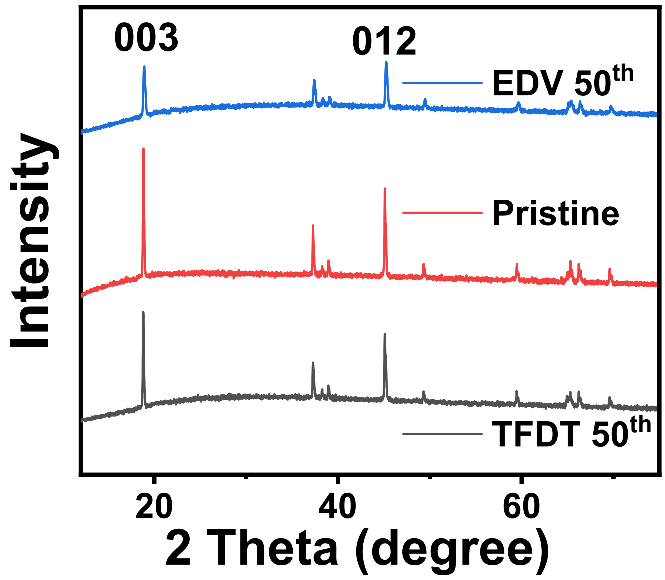

Fig. S1 XRD spectra of LCO from different electrolytes before and after cycling.

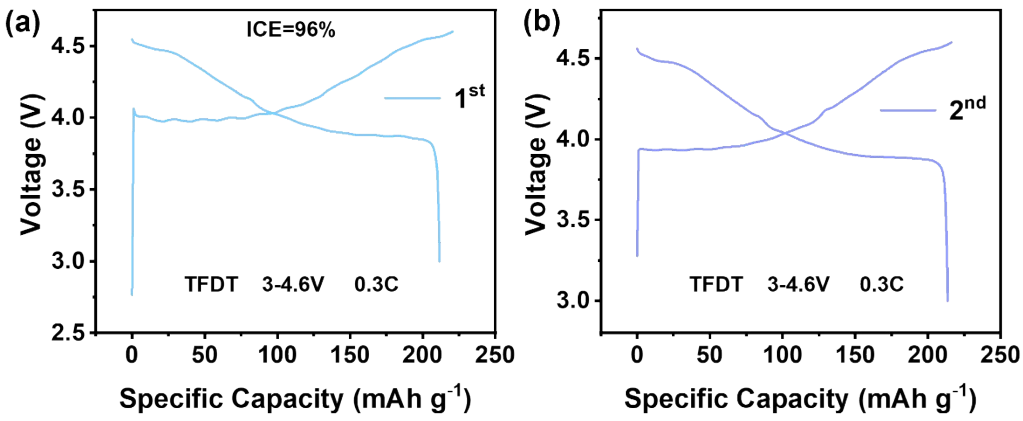

Fig. S2 Charge-discharge curves of Li||LCO cells with the TFDT electrolyte at 0.3 C for the first and second cycle in the voltage range 3-4.6 V.

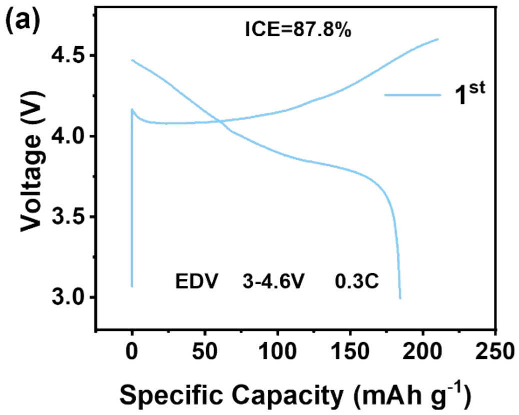

Fig. S3 Charge-discharge curves of Li||LCO cells with the EDV electrolyte at 0.3 C for the first and second cycle in the voltage range 3-4.6 V.

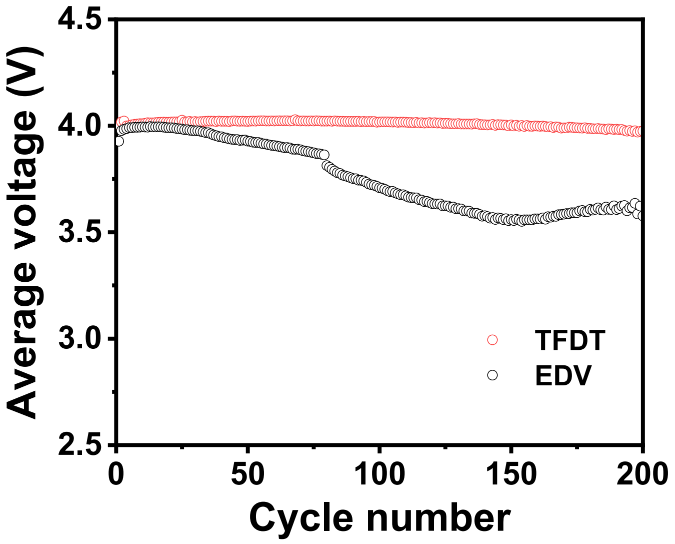

Fig. S4 Average discharge voltage of LCO||Li cell as a function of cycle number.

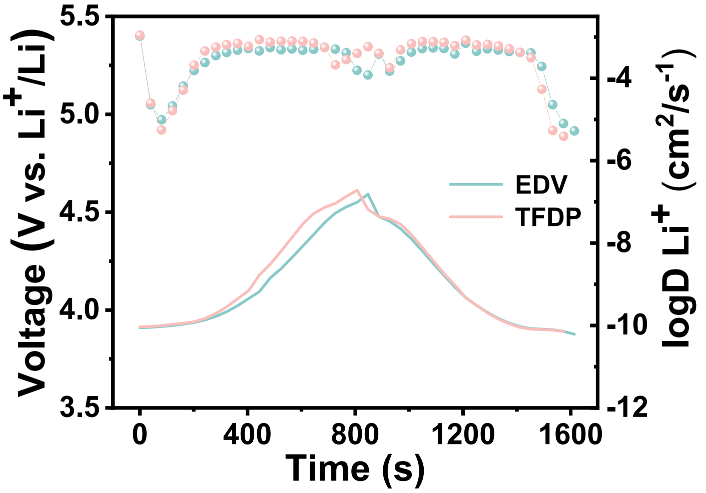

Fig. S5 Galvanostatic intermittent titration test curve of EDV and TFDT electrolytes.

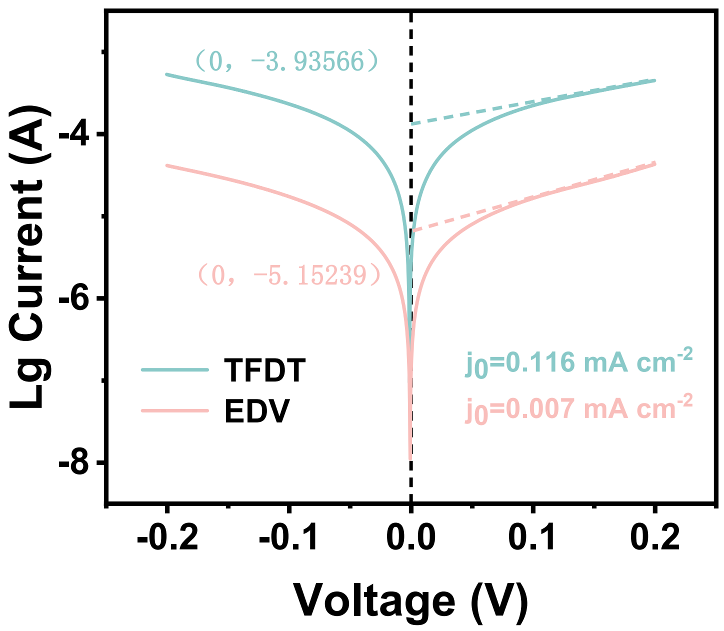

Fig. S6 Tafel plots of symmetric Li cells in different electrolytes.

4. Supplementary tables

Table S1. The relative peak intensities of LCO cathodes after different electrolyte cycles

Electrolyte	003(18.9°)	012(44.9°)
TFDT	0.389	0.526
EDV	0.742	0.765
Pristine	1	1

Table S2. Summary of the roughness parameters of lithium metal surfaces after 50 cycles of different electrolytes

Electrolyte	R_q (nm)	R_a (nm)
TFDT	3.95	2.01
EDV	38.7	28.7

Table S3. Summary of various parameters from Cone calorimeter experiment

Electrolyte	CO ratio (kg/kg)	CO_2 ratio (kg/kg)
TFDT	0.0124	0.1
EDV	0.005	0.01
